# Perinatal Gene-Gene and Gene-Environment Interactions on IgE Production and Asthma Development

**DOI:** 10.1155/2012/270869

**Published:** 2012-02-28

**Authors:** Jen-Chieh Chang, Lin Wang, Rong-Fu Chen, Chieh-An Liu

**Affiliations:** ^1^Institute of Biomedical Sciences, National Sun Yat-Sen University, Kaohsiung 804, Taiwan; ^2^Genomic and Proteomic Core Laboratory, Department of Medical Research, Kaohsiung Chang Gung Memorial Hospital and Chang Gung University College of Medicine, Kaohsiung 833, Taiwan; ^3^Department of Pediatrics, Kaohsiung Chang Gung Memorial Hospital and Chang Gung University College of Medicine, Kaohsiung 833, Taiwan; ^4^Department of Medical Research, Chang Bing Show Chwan Memorial Hospital, Changhua 505, Taiwan

## Abstract

Atopic asthma is a complex disease associated with IgE-mediated immune reactions. Numerous genome-wide studies identified more than 100 genes in 22 chromosomes associated with atopic asthma, and different genetic backgrounds in different environments could modulate susceptibility to atopic asthma. Current knowledge emphasizes the effect of tobacco smoke on the development of childhood asthma. This suggests that asthma, although heritable, is significantly affected by gene-gene and gene-environment interactions. Evidence has recently shown that molecular mechanism of a complex disease may be limited to not only DNA sequence differences, but also gene-environmental interactions for epigenetic difference. This paper reviews and summarizes how gene-gene and gene-environment interactions affect IgE production and the development of atopic asthma in prenatal and childhood stages. Based on the mechanisms responsible for perinatal gene-environment interactions on IgE production and development of asthma, we formulate several potential strategies to prevent the development of asthma in the perinatal stage.

## 1. Introduction

Atopic asthma is a complex disease associated with IgE-mediated allergic reactions. Most allergens elicit IgE antibodies, which bind to mast cells; when cross-linked, the mast cell releases inflammatory mediators that cause bronchospasm and mucus formation [1]. Nonatopic asthma refers to inflammation and constriction of the airways that are not caused by exposure to an allergen. As with numerous ill-defined diseases in which numerous extrinsic influences and genetic factors contribute to onset of the disease, the term “complex disease” is applied. Such terminology refers to asthma as caused by a complex relationship between genetic and environmental components, resulting in the clinical manifestations of atopic asthma. Systemic administration of humanized anti-IgE antibodies causes a 95–99% decrease in serum IgE, along with anti-inflammatory feature of asthma [2], which supports the IgE-mediated mechanism of atopic asthma. Numerous genome-wide association (GWA) studies have identified more than 100 genes in 22 chromosomes associated with atopic asthma [3, 4]. Moreover, genetic backgrounds and environmental exposures could modulate susceptibility to asthma [5, 6]. This suggests that asthma, although heritable, is significantly affected by environmental factors. Evidence has recently shown that molecular mechanisms of atopic disease may not be limited to DNA sequence differences, but also gene-environmental interactions for epigenetic difference and/or regulatory T cells (Treg) [7, 8]. We describe recent advances in our understanding based on the mechanisms responsible for gene-gene and gene-environment interactions on IgE production and development of atopic asthma in the perinatal stage.

## 2. Gene-Gene Interactions on IgE Production and Asthma Development

Allergic diseases, including atopic asthma, have long been attributed to IgE-mediated reactions, and elevation of serum IgE levels has been correlated to allergic diseases [29, 30]. Allergic sensitization might occur *in utero* and be related to the future development of allergic diseases [31, 32]. Elevation of cord blood IgE (CBIgE), although not sufficient to predict the development of allergic diseases in childhood [33, 34], was shown to be a risk predictor for the development of aeroallergen sensitization [35] and for later development of childhood asthma [36]. Significant associations with elevation of CBIgE levels were reported previously for *cytotoxic T-lymphocyte-associated protein 4 *(*CTLA4*) +49A allele [37] and for *IL13, CCL17,* and *CXCL10* gene interactions [10].

Reijmerink et al. [11] used the multifactor dimensionality reduction (MDR) analysis, which is designed to translate high-dimensional genetic data into a single dimension, to explore the gene-gene interactions on IgE production and the development of asthma. Interactions between these genes, located in the Toll-like-receptors- (TLR-) related pathway, showed that the polymorphisms in *interleukin 1 receptor-like 1 *(*IL1RL1*) and *bactericidal/permeability-increasing protein *(*BPI*) were the optimal model of interaction using 2-way MDR analysis (*P* = 0.02) to predict the elevation of total IgE levels. The polymorphisms in *myeloid differentiation factor *(*MD*)*-2, beta-2 integrin *(*ITGB2*), and *BPI* were identified as the optimal model of the 3-way MDR analysis (*P* = 0.01) to predict the elevation of total IgE levels at 1 to 2 years of age.

A number of gene-gene interactions implicating a link between IgE production and the development of asthma in the perinatal stage are shown in Table 1, in which more complex interactions among different immune genes are found in asthma than in IgE production. Moreover, a kinetic change of different gene profiles associated with IgE production was found in children with increasing ages. In our studies on Chinese cohorts, IgE production in infancy and toddlerhood was associated with immune and remodeling genes, and IgE production in preschool age was associated with MHC class II antigen genes, such as *HLA-DPA1* and *HLA-DQA1 *(our unpublished data). These results suggest that altered immune remodeling in infancy and toddlerhood may prime children for allergic sensitization in childhood, depending on HLA genotypes.

With the introduction of powerful novel genetic-analysis tools, the heritable component of asthma has gained increasing attention over the last decade. This attention calls for open approaches to the linkage and GWA studies rather than traditional candidate gene approaches to the genetics of asthma. The main strength of GWA studies lies in their ability to discover genuinely novel disease-candidate genes, especially those associated with moderate risks [38]. In a recent GWA study for asthma, Moffatt et al. genotyped more than 317,000 single-nucleotide polymorphisms (SNPs) in 994 patients with childhood asthma and 1243 people without asthma, and identified that polymorphisms of* ORM1-like 3* (*ORMDL3*) on chromosome 17q21.1 were strongly associated with childhood asthma [39]. The association was also independently replicated in 2320 participants from a cohort of German children and in 3301 participants from the British 1958 birth cohort [39].

*ORMDL3* appears to be a gene in a very old part of the human genome, and similar genes were found in primitive organisms such as yeast. Although the transcript level of *ORMDL3* is strongly correlated to susceptibility to childhood asthma, its role remains unclear. Additional GWA studies on asthma are underway, and cross-validation data among these studies may lead to better conclusions on the responsible genes for the development of asthma.

## 3. Gene-Environment Interactions on IgE Production and Asthma Development

Increasingly, more studies in the literature identify novel genes associated with asthma and suggest that numerous genes with small effects rather than few genes with strong effects contribute to the development of asthma. These genetic effects may in part differ with respect to a patient's environmental exposures. Several environmental factors, such as maternal atopy, endotoxin, tobacco smoke, pollutants, allergens, cold air, microbial infections, medication, and exercise, are known to exacerbate asthma symptoms [40–44]. Of these environmental factors, maternal atopy, microbial exposure, and tobacco smoke exposure are particularly important and its gene-environment interactions are described below.

### 3.1. Maternal Atopy

Allergy sensitization may occur in fetal life. Moreover, the immature immune system is highly susceptible to immunomodulatory environmental conditions, particularly in the prenatal and postnatal periods [45]. Maternal atopy may impact neonatal immune development and subsequently alter the allergic responses of neonates. A number of candidate gene studies investigating interactions between maternal atopy and airway hyperresponsiveness have also been performed for 3 main groups of genes: immunity genes (*CD14, IL13, CCL22,* and *CTLA4*), the stress gene (*fibroblast growth factor 1* (*FGF-1*)), and the MHC class I antigen gene (*HLA-G*) gene. Specifically, maternal but not paternal atopy has a significant impact on CBIgE elevation, depending on the gender and *CTLA4* +49A/G polymorphism of newborns [46]. Maternal atopy interacting with the polymorphisms in *IL13* and *CCL22* genes was reported to be a good predictor of CBIgE elevation [10]. In contrast, in the absence of maternal atopy, the *CTLA4* +49GG genotype in female newborns had a protective effect on CBIgE elevation [46].

### 3.2. Microbial Exposure

The prevalence of asthma is widely skewed in developed and developing countries, the reasons for which remain obscure. The hygiene hypothesis has emerged as a popular explanation. The hygiene hypothesis was initially developed to explain a reduced rate of allergic diseases among children with older siblings [56]. The underlying concept is that an increased exposure to microbes and their products during the perinatal stage protects against the development of allergic responses to common environmental antigens, such as dust mites, fungi, cockroaches, and pollens. Although abundant epidemiological studies support the hygiene hypothesis, significant inverse relations between exposures to these compounds and atopy and asthma have been found.

Protection from allergies is conferred by children growing up on small farms in parts of Europe [57], supporting this hypothesis. The specific exposure responsible for the protection against allergies afforded by this rural lifestyle is unknown, but most of the results have focused on germs and their endotoxin, which can be measured in house dust. Endotoxin, a component of the cell walls of Gram-negative bacteria, is one of the pathogen-associated molecular patterns (PAMPs). PAMPs, evolutionarily highly conserved structural components of microbes, are recognized by conserved receptors of innate immune systems, pattern-recognition receptors (PRRs), the human CD14, TLRs, nucleotide-binding oligomerization domain (NOD)-1, NOD2, and C-type lectin receptors. Because the specific agent responsible for the protective effect against asthma in children with a rural upbringing is unknown, the exposure metric in some studies on gene-environment interaction on IgE levels, allergies, or asthma in children growing up on farms relies on self-reported contact with germs and other animals, as listed in Table 2.

The relationship between endotoxin exposure and IgE levels seems to be affected by a common SNP in the promoter region of *CD14*, a receptor involved in endotoxin recognition [50]. A significant gene-environment interaction exists between the *CD14* −260C/T genotype and endotoxin exposure on CD4^+^ lymphocyte numbers, particularly CD4^+^Foxp3^−^ lymphocytes at 1 year of age [47]. Interactions of *IL13* with *IL4R*, *IL13R*, or *CCL17*, as well as the interactions of *CD14* with daycare attendance, endotoxin, or rural living affect IgE production and the development of asthma in childhood [14–17, 21, 22, 50–52].

### 3.3. Tobacco Smoke Exposure

 Previous studies have provided evidence on the role of tobacco smoke exposure (TSE) as a determinant risk factor of childhood asthma [40, 75, 76]. Recent studies suggest that *in utero* TSE from maternal smoking during pregnancy is associated with reduced lung function and constitutes a significant risk factor for the development of asthma [75, 77–81]. This parent-of-origin effect points to a significant role of the maternal prenatal environment on later asthma risk in the offspring [82]. Early postnatal TSE from parents was also associated with infant wheezing and lower respiratory tract infections and increased the prevalence of asthma independently from the effect of *in utero *TSE from maternal smoking during pregnancy [83]. Moreover, the effect of current TSE is known not only to influence the severity of asthma, but also to impair the efficacy of inhaled corticosteroid treatments [84]. Although exposure to active and passive cigarette smoking is a well-recognized risk factor for the development of asthma, asthma manifests in only a portion of people [85, 86]. Furthermore, Jaakkola et al. showed that a combination of parental atopy and TSE has a synergistic effect on the risk of childhood asthma [85]. This suggests that the effect of environmental factors on the development of asthma can be modified by genetic constitution.

A number of candidate gene studies investigating interactions with TSE have been performed for 3 main groups of genes: innate-immunity genes (*CD14, tumor necrotic factor *(*TNF*), and* IL-1 receptor antagonist *(*IL1RN*)), adaptive-immunity genes (*IL13* and *transforming growth factor *(*TGF*)*-*β*1*), and response and remodeling genes (*a disintegrin and metalloprotease domain-containing protein* (*ADAM*)-33, *glutathione S-transferase* (*GST*)-*M1, GSTP1*, and **β*2-adrenergic receptor* (*B2AR*)), as shown in Table 3. Timing of exposure to TSE is also an important consideration in the analysis of gene-environment interactions on the development of childhood asthma. In the prenatal stage, the *IL1RN* gene polymorphism rs2234678 GG genotype significantly increased the relative risk of asthma only in children of mothers who smoked during pregnancy [63]. Reijmerink et al. showed that *ADAM33* polymorphisms increased the risk of developing asthma *in utero*, but postnatal TSE did not [59]. A GWA study demonstrated that the chromosomes 3p and 5q were linked to childhood asthma in tobacco-exposed families [67]. Wang et al. [69] showed that the joint effect of the *B2AR* polymorphism at position 16 with TSE increased the risk of asthma in a dose-dependent manner. Some genes were only associated with asthma in the presence of TSE [63, 64]. In *GSTM1*-null children of school age, *in utero* exposure to smoking is associated with an increased prevalence of early-onset asthma, asthma with current symptoms, persistent asthma, lifetime history of wheezing, wheezing with exercise, wheezing requiring medication, and number of emergency department visits in the past year in comparison to children with the *GSTM1^+^* genotype [61, 87, 88].

A recent study showed a synergistic effect of air pollution levels and functional SNPs within catalase and myeloperoxidase on the respiratory-related school absence of asthmatic children [74]. The exposure-traffic air pollutants also increased the effect of *TGFB1* −509C/T polymorphism on the development of asthma [66].

## 4. Environmental Influence of Epigenetic Programming for IgE Production and Asthma Development in the Postnatal Stage

Epigenetic programming is broadly defined as heritable changes in gene expression or cellular phenotype other than changes in DNA sequences [95, 96]. Epigenetic alterations are believed to occur not only prenatally or shortly after birth, but also during later developmental periods, influencing gene expression differentially throughout the lifespan. Although GWA studies hold promise for identifying unexpected gene-environment interactions, how the gene-environment interactions affect IgE production and asthma development remains unclear. One of the potential mechanisms is the epigenetic programming of asthma by gene-environment interactions in the perinatal stage. Early-life dietary supplementation and environmental exposures are known to affect adult metabolism and phenotype through alterations in DNA CG methylation [97].

Several environmental exposures reportedly interact with genetic predisposition through epigenetic mechanisms on total IgE levels and asthma phenotype (Table 4). Hollingsworth et al. are the first to report an increased risk of allergic disease in *in utero *dietary methyl donors because of the differential methylation of 82 genes [90]. One of these is *runt-related transcription factor 3* (*Runx3*), a gene known to down*­*regulate allergic airway inflammation; it is associated with decreased transcriptional activity and mRNA expression in lung tissue. DNA methylation levels of *Runx3* increased in animals exposed to methyl donors *in utero* [90]. This effect could be transmitted to subsequent progeny mice. *Runx3* is known to cooperate with T-bet in the silencing of IL-4 in Th1 cells [98]. A reduced Runx3 level might lead to enhanced transcription of IL-4 skewing toward Th2 differentiation, as observed in anti-CD3^+^CD28^+^ antibody-stimulated CD4^+^ lymphocytes. Although the significance of epigenetic inheritance in humans is unclear, one recent study reported that folate supplements in pregnancy are associated with increased childhood wheezing [92]. In contrast, 2 cohort studies showed that dietary pattern during pregnancy was not associated with recurrent wheezing [99] or asthma and related outcomes [100].

Maternal smoking in pregnancy may feasibly impact the development of allergic airway disease through epigenetic pathways because of changes in DNA methylation or histone modifications. Air pollution has been also linked to epigenetic changes in *ACSL3* [65]. Similarly, diesel exhaust particles were shown to affect CG methylation of *IL4* and *IFNG* promoters, which are involved in airway inflammation [91].

## 5. Implications of Gene-Environment Interactions on Prevention of Asthma

**Figure 1:**
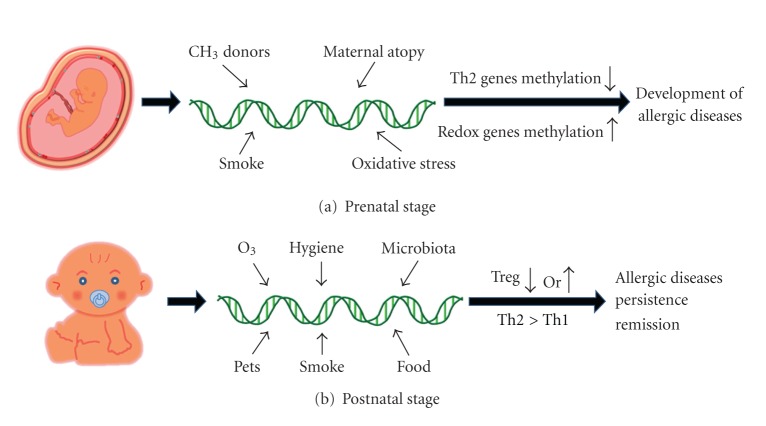
Mechanisms of pre- and postnatal environmental factors on the development of asthma. A number of prenatal factors such as maternal diet with methyl donors, maternal atopy, TSE, and oxidative stress could alter the epigenetic programming of Th2 and redox genes, resulting in the allergic sensitization and diseases (a). Additional postnatal environmental conditions such as pollution, secondhand TSE, pets exposure, infant diets and microbiota may modulate or drive the expression of Treg and/or Th1/Th2 genes resulting in skewed or balanced Th2 responses that contribute to persistence or remission of allergic diseases (b).

IgE production and asthma are not controlled by a single gene, but are involved in a complex interaction with environmental modification of genetic and epigenetic programming of asthma. Any individual study is unlikely to be able to account for all of the complex interactions with confounding factors in prenatal and postnatal stages and from other host factors such as maternal diets, obesity, and gender. Despite these limitations, the study of environmental epigenetics promises to help us understand the theoretically preventable disease, asthma. Environmental changes can epigenetically modulate Th2 and redox genes in the prenatal stage and change the Treg function and/or skewed Th2 immune reaction in the postnatal stage, as shown in Figure 1. Notable differences in the immune responses of allergic and nonallergic children are evident *in utero*, where environmental exposures such as maternal diet with methyl donors, maternal atopy, TSE, and oxidative stress could alter the epigenetic programming of Th2 and redox genes, resulting in allergic sensitization and diseases (Figure 1(a)). Additional postnatal environmental conditions such as pollution, secondhand TSE, pet exposure, infant diets, and microbiota may modulate or drive the expression of Treg and/or Th1/Th2 genes, resulting in skewed or balanced Th2 responses that contribute to persistence or remission of allergic diseases (Figure 1(b)). Based on the mechanisms responsible for the perinatal epigenetic and immune regulation of the development and remission of allergic diseases, we may be able to formulate potential strategies to prevent the development of asthma in the perinatal stage by manipulating perinatal conditions such as diet control or complementary food and by early screening of DNA methylation changes followed by modulation of CG methylation levels.

## 6. Conclusion

Increasingly, more evidence suggests that different gene-environment interactions play an important role on IgE production before and after birth. We conducted a systematic review of recent studies to identify the roles of gene-gene and gene-environmental interactions on the prenatal and childhood IgE production, as well as the development of asthma. Prevention of IgE production and IgE-mediated diseases may be possible by controlling different environmental factors for patients with susceptible genotypes early in the perinatal stage. Control of maternal atopy in pregnancy and modulation of gene expression such as *CTLA4 *and *IL13* may be a target for decreasing antenatal IgE production and possibly lowering perinatal allergy sensitization. TSE is a well-recognized risk for the development of childhood asthma; reducing the perinatal exposure of tobacco smoke may prevent the development of asthma, particularly in subjects carrying susceptible Th2 and redox genotypes. Further studies are necessary to compare gene-gene and gene-environment interactions on IgE production and asthma development in different ethnic populations and to study whether manipulations of maternal diets or postnatal complementary food could modulate epigenetic programming of asthma.

##  Author's Contributions

Jen-Chieh Chang and Lin Wang contributed equally to this study.

## Figures and Tables

**Table 1:** Gene-gene interactions on IgE production and asthma phenotype.

Total IgE levels/ asthma phenotype		Gene-gene interactions
Prenatal	IgE levels (cord blood, infant blood)	*CD86*-*VTCN1 *[9]
		*IL13*-*CCL17 *[10]
		*IL1RL1*-*BPI *[11]
		*TGFBR2*-*IL2RA *[12]
		*IL13*-*CCL17*-*CXCL10 *[10]
		*MD2*-*ITGB2*-*BPI *[11]

Childhood	IgE levels	*CD14*-*AOAH *[13]
		*IL1RL1*-*NOD1 *[11]
		*IL13*-*IL4RA *[14, 15]
		*IL13*-*IL13RA1 *[16]
		*IL13*-*TARC *[17]
		*TLR2*-*TGFBR2 *[12]
		*MD2*-*MAP3KIP2*-*BPI *[11]
		*TLR2*-*IL6RA*-*IL2RA *[12]
	
	
	
	Asthma	*CD14*-*AOAH *[13]
		*CD274*-*LILRA4 *[9]
		*GSNOR*-*B2AR *[18]
		*IL4-IL4RA *[19]
		*IL6*-*IL6R *[20]
		*IL13*-*IL4R *[21]
		*IL13*-*IL4RA *[15, 17, 22]
		*LTA4H*-*ALOX5AP *[23]
		*SOCS1*-*MAP3K7IP1 *[11]
		*TNC*-*NPSR1 *[24]
		*EPHX1*-*CYP1B1*-*CYP2D6 * [25]
		*IL2RA*-*FOXP3*-*IL2RA *[12]
		*IRAK1*-*NOD1*-*MAPK7IP1 * [11]
		*STAT6*-*STAT4*-*IFNG *[26]
		*TLR2*-*IL2RA*-*TGFBR2 *[12]
		*B2AR*-*CCR3*-*CysLTR1*- *FCER1B *[27]
		*INSIG2*-*IL4*-*CHIA*-*ADIPOQ*- *ALOX5 *[28]
		*IL4*-*STUB1*-*ADRB2*-*IL4RA*- *IL13RA2*-*CHIA *[28]

**Table 2:** Interactions of genes with maternal atopy, endotoxin and other environmental factors on IgE production and asthma phenotype.

		Environment factors-gene interaction
Prenatal	Cord blood IgE levels	Maternal atopy-*FGF1 *[10]
		Maternal atopy-*IL13*-*CCL22* [10]
		Maternal atopy-gender-*CTLA4* [46]
		Endotoxin-*CD14 *[47]

Childhood	IgE levels	Animal contact-*CD14 *[48]
		Helicobacter pylori-*CD14 *[49]
		Endotoxin-*CD14 *[50]
		Day care attendance-*CD14 *[51]
		Day care attendance-*IL4R* [51]
	
	
	
	Asthma	Country living-*CD14* [52]
		Fungi-*CHIT1 *[53]
		Maternal bronchial hyperresponsiveness-*HLA-G* [54]
		Children of farmer-*TLR2* [55]

**Table 3:** Interactions of genes with perinatal environmental TSE and pollution on asthma development.

Stage	Environment factors-gene interaction
Prenatal	TSE-Chromosome 1q43-q44, 4q34 and 17p11 [58]
	TSE-*ADAM33 *[59]
	TSE-*B2AR *[60]
	TSE-*GSTM1 *[5, 61]
	TSE-*GSTP1 *[62]
	TSE-*IL1RN *[63]
	TSE-*IL13 *[64]
	Pollution-*ACSL3 *[65]
	TSE and pollution-*TGFB1 *[66]

Childhood	TSE-Chromosome 3p and 5q [67]
	TSE-Chromosome 1p, 1q, 5q, 9q and 17p [68]
	TSE-Chromosome 1q43-q44, 4q34 and 17p11 [58]
	TSE-*B2AR *[69]
	TSE-*CD14 *[70]
	TSE-*GSTP1 *[71]
	TSE-*GSTM1 *[71]
	TSE-*TNF *[72]
	Pollution-*GSTM1*-*TNF *[73]
	Pollution-*GSTP1*-*TNF* [73]
	Pollution-Catalase genes-Myeloperoxidase genes [74]

**Table 4:** Environmental modification of epigenetic program on IgE production and asthma.

Phenotypes	Environment factors-epigenetic modification
IgE levels	High-fat (HF) intake-obesity [89] *murine *
	Maternal diet with methyl donors-82 loci [90] *murine *
	Aspergillus fumigatus-diesel exhaust particles (DEP)-*IL4* promoter [91] *murine *
	Aspergillus fumigatus-diesel exhaust particles (DEP)-*IFNG* promoter [91] *murine *

Asthma	Folic acid supplements in pregnancy [92]
	A lwoffii F78-*IFNG* promoter [93] *murine *
	Maternal diet with methyl donors-82 loci [90] *murine *
	Pollution-*ACSL3 *[65]
	Microbial exposure-*IFNG *[94]
